# A Proof-of-Concept Pilot Study of Contrast-Enhanced Ultrasound as a Potential Alternative to Contrast-Enhanced Magnetic Resonance Imaging in the Surveillance of Hepatocellular Adenoma and Focal Nodular Hyperplasia

**DOI:** 10.3390/biomedicines14020437

**Published:** 2026-02-15

**Authors:** Adam Dobek, Mateusz Kobierecki, Adam Fabisiak, Wojciech Ciesielski, Marta Lenk-Jędrzejczak, Filip Franciszek Karuga, Filip Andrzej Dąbrowski, Ewa Małecka-Wojciesko, Ludomir Stefańczyk

**Affiliations:** 1 I Department of Radiology and Diagnostic Imaging, Norbert Barlicki Memorial Teaching Hospital No. 1, Medical University of Lodz, 90-153 Lodz, Poland; 2 Department of Diagnostic Imaging, Polish Mother’s Memorial Hospital Research Institute, 93-338 Lodz, Poland; 3 Department of Digestive Tract Diseases, Norbert Barlicki Memorial Teaching Hospital No. 1, Medical University of Lodz, 90-153 Lodz, Poland; 4 Department of General Surgery and Transplantology, Norbert Barlicki Memorial Teaching Hospital No. 1, Medical University of Lodz, 90-153 Lodz, Poland; 5 Department of Gynecology and Gynecological Oncology, Centre of Postgraduate Medical Education CMKP, 01-813 Warsaw, Poland; 6 Department of Sleep Medicine and Metabolic Disorders, Medical University of Lodz, 90-419 Lodz, Poland

**Keywords:** hepatocellular adenoma, focal nodular hyperplasia, contrast-enhanced, ultrasound, ultrasonography, magnetic resonance imaging

## Abstract

**Background**: Focal nodular hyperplasia (FNH) and hepatocellular adenoma (HA) are benign hepatic tumors that predominantly affect women of reproductive age and are associated with hormonal and metabolic factors. While FNH is a non-progressive lesion without malignant potential, HA carries a relevant risk of hemorrhage and malignant transformation. Differentiation between these entities remains challenging due to overlapping imaging features. Although contrast-enhanced magnetic resonance imaging (MRI) is considered the diagnostic reference standard, its cost, limited availability, and contraindications restrict routine long-term use. Therefore, contrast-enhanced ultrasound (CEUS) has emerged as an alternative modality for follow-up. This study evaluated the effectiveness of CEUS in long-term monitoring of FNH and HA compared with MRI. **Methods**: Patients with imaging-confirmed FNH or HA underwent paired CEUS and MRI examinations within 48 h at baseline and follow-up. Lesion size was assessed using maximal and minimal diameters, and longitudinal changes were classified according to RECIST-like criteria. Paired non-parametric statistical tests were applied. **Results**: 41 benign liver lesions (28 FNH and 13 HA) were analyzed across 92 paired examinations. Baseline lesion measurements were comparable between CEUS and MRI. A statistically significant difference was observed in the assessment of the largest lesion diameter, while no significant differences were detected for the shortest diameter. Longitudinal evaluation showed no significant differences between modalities in detecting lesion size changes. Response classification was concordant in 42 of 51 follow-up assessments, with stable disease as the most frequent outcome. **Conclusions**: After definitive diagnosis, CEUS may serve as a reliable standalone modality for routine long-term surveillance of FNH and HA in clinically stable patients. Its performance in lesion measurement and response assessment is comparable to MRI, while offering advantages in cost, accessibility, and patient tolerability. MRI may be reserved for cases with suspicious changes on CEUS.

## 1. Introduction

Focal nodular hyperplasia (FNH) is the second most common benign hepatic neoplasm, accounting for approximately 8% of primary liver tumors. Hepatocellular adenoma (HA), although significantly less prevalent, is associated with a substantially higher risk of clinical complications. Both entities predominantly affect women, with a reported female-to-male ratio of up to 9:1, and are most frequently diagnosed in women of reproductive age, particularly those using hormonal contraceptives. Additional risk factors identified in the literature include androgen therapy, metabolic disorders such as glycogen storage disease and maturity-onset diabetes of the young type 3, obesity, chronic alcohol use, and conditions associated with endocrine dysregulation, including Klinefelter syndrome and polycystic ovary syndrome. Certain pharmacologic agents, such as clomiphene and barbiturates, have also been implicated in the pathogenesis of these lesions [1,2,3,4]. HA carries a recognized risk of spontaneous rupture, potentially resulting in intra-abdominal hemorrhage, as well as malignant transformation to hepatocellular carcinoma (HCC). The incidence of hemorrhagic complications has been reported to be as high as 20%, while the risk of malignant progression is estimated at approximately 5%, with both risks markedly increased in lesions exceeding 50 mm in diameter [4,5,6,7,8,9]. In contrast, FNH is considered a benign, non-progressive lesion without malignant potential and is typically managed conservatively. Although FNH itself does not require oncologic surveillance, follow-up imaging may be warranted in selected cases to confirm diagnostic stability and exclude misclassification with HA [10,11,12,13,14]. Accurate diagnosis and appropriate long-term monitoring of benign hepatic tumors are therefore essential, particularly to guide management decisions and to ensure diagnostic stability over time. Advanced imaging modalities, especially contrast-enhanced magnetic resonance imaging (MRI) and contrast-enhanced ultrasound (CEUS), play a central role in the diagnosis and follow-up of focal liver lesions [5,6,15,16]. Although MRI is widely regarded as the diagnostic gold standard due to its strong concordance with histopathological findings, its routine use is limited by high cost, restricted availability, and relevant contraindications [15,16,17]. CEUS represents a more accessible, cost-effective, and well-tolerated alternative with a favorable safety profile [5,6,18]. However, robust data supporting its role in long-term surveillance are still limited, and direct comparisons with MRI remain scarce. Therefore, this study aimed to evaluate the effectiveness of CEUS in the long-term monitoring of FNH and HA, with particular emphasis on its performance relative to MRI.

## 2. Materials and Methods

### 2.1. Study Group

The study was conducted in accordance with the Declaration of Helsinki and received approval from the local Bioethics Committee (RNN/266/22/KE). Written informed consent was obtained from all participants prior to enrollment. Adult patients (≥18 years) with a confirmed diagnosis of HA or FNH were eligible for inclusion provided that the diagnosis was established on the basis of concordant MRI and CEUS findings, in line with EASL guidelines allowing non-histological diagnosis when two imaging modalities demonstrate definitive agreement [19]. Subcentimeter lesions (<10 mm) were included primarily as satellite nodules in multifocal cases, where they exhibited enhancement patterns identical to a larger, definitive index lesion. This approach allowed for the long-term assessment of whether these benign vascular patterns might evolve to demonstrate features typical of malignancy, consistent with our previously validated methodology [5,6]. CEUS and MRI were performed within a maximum interval of 48 h. Eligibility additionally required the availability of at least one follow-up assessment, defined as paired baseline and follow-up CEUS–MRI examinations, with a minimum interval of six months between assessments. Exclusion criteria comprised contraindications to ultrasound contrast agents (SonoVue, Bracco Imaging S.p.A., Milan, Italy), including respiratory insufficiency, acute coronary syndrome, prior hypersensitivity reactions, or pregnancy. For analytical purposes, focal liver lesions rather than individual patients were considered the unit of analysis. Overall, 32 patients underwent 184 paired MRI–CEUS examinations, which were organized into 92 longitudinal observations, each consisting of a baseline and a follow-up MRI–CEUS pair. This study design enabled a direct comparison of the long-term performance of CEUS and MRI in the evaluation and surveillance of benign liver lesions. Detailed demographic characteristics are presented in the Results section.

### 2.2. Magnetic Resonance Imaging Protocol

MRI examinations were performed using 1.5 Tesla or 3.0 Tesla scanners (Siemens Magnetom Avanto and Siemens Magnetom Vida, Siemens Healthineers AG, Erlangen, Germany) with phased-array body coils. All patients fasted for at least six hours prior to the scan. Informed consent was obtained, and renal function was evaluated to confirm eligibility for contrast administration. MultiHance (gadobenate dimeglumine) was administered intravenously at a dose of 0.2 mL per kilogram of body weight (equivalent to 0.1 mmol/kg), followed by a 20 mL saline flush at a rate of 1 mL/s. The imaging protocol included axial T1-weighted in-phase and opposed-phase gradient echo sequences, axial and coronal T2-weighted images with and without fat suppression, fat-suppressed axial T2-weighted images (optionally with respiratory triggering), and diffusion-weighted imaging with multiple b-values (0, 50, 400, and 800 s/mm^2^) and apparent diffusion coefficient maps. Dynamic post-contrast imaging was performed using fat-suppressed 3D T1-weighted gradient echo sequences. Arterial phase images were acquired 15–25 s after contrast injection using either bolus tracking or a fixed delay, followed by portal venous phase (60–70 s) and delayed phase (3–5 min). No hepatobiliary phase imaging was performed. Typical parameters for dynamic sequences included a repetition time of approximately 4.5 ms, echo time of 2.2 ms, flip angle of 10–15°, slice thickness of 3 mm, and field of view adapted to patient body size. All sequences were acquired during breath-hold with parallel imaging techniques.

### 2.3. Contrast-Enhanced Ultrasound Protocol

CEUS examinations were performed in accordance with the 2020 updated guidelines for contrast-enhanced ultrasound of the liver [20]. A GE Logiq 7 system equipped with a 4C convex probe was used. The procedure began with a standard grey-scale (B-mode) ultrasound to assess liver anatomy and identify any lesions that might not demonstrate contrast enhancement, which could otherwise be misinterpreted as malignant. The number, size, and location of all detected lesions were recorded. Color Doppler imaging was then conducted to evaluate vascular patterns. Subsequently, CEUS was performed following the intravenous administration of 2.4 mL of the contrast agent SonoVue into the medial cubital vein. In most cases, this dose was sufficient to achieve diagnostic clarity; however, an additional dose was administered when necessary. The examination was conducted using a low mechanical index (<0.1) to preserve the integrity of microbubble contrast agents [21,22]. Three dynamic vascular phases were assessed: the arterial phase (10–45 s post-injection), the portal venous phase (45–120 s), and the late venous phase (120–180 s) [20]. No significant contrast enhancement changes were observed beyond 120 s. In cases where lesions were located in subdiaphragmatic regions, deep inspiration and breath-holding were required, which posed difficulties for some patients and limited the feasibility of extended acquisitions. Therefore, extended acquisition beyond 180 s was reserved for inconclusive cases only. Throughout the examination, lesion enhancement patterns were evaluated in comparison to the surrounding liver parenchyma.

### 2.4. Statistical Analysis

To increase the statistical power of the analyses, HA and FNH were analyzed as a single study group, as both entities exhibit comparable enhancement patterns on MRI and CEUS [5,6]. Lesions were evaluated longitudinally using repeated measurements obtained from sequential imaging examinations, with each lesion directly compared with its prior assessment, enabling paired within-lesion analyses. All measurements were performed in the arterial phase on both MRI and CEUS examinations and were independently reviewed by two radiologists, with final approval reached by consensus. In cases of disagreement, a third radiologist with more than 30 years of experience in abdominal radiology was consulted to resolve discrepancies. Statistical analyses were conducted using Python (version 3.14) with the pandas, SciPy, and statsmodels libraries. Continuous variables are reported as medians with interquartile ranges (IQR). To account for the non-independence of repeated measurements within the same lesion (clustering), linear mixed-effects models were employed to compare lesion diameters and their relative changes between CEUS and MRI. In these models, the imaging modality was treated as a fixed effect, while the unique lesion identifier was included as a random effect to account for the hierarchical structure of the data. The relative change was calculated for each follow-up interval as the ratio of the sum of the longest and shortest diameters at the current examination to the corresponding sum from the preceding assessment. A *p*-value < 0.05 was considered statistically significant. Changes in lesion size over time were assessed using criteria analogous to the Response Evaluation Criteria in Solid Tumors (RECIST), version 1.1. Although RECIST was originally developed for the evaluation of malignant tumors and is not formally applicable to benign lesions, this approach was applied analogously in accordance with previously published studies on long-term imaging follow-up of benign liver lesions, including that by Vernuccio et al. [9,23]. Lesion regression was defined as complete disappearance of the lesion or a decrease of at least 30% in lesion diameter compared with baseline. Lesion progression was defined as the appearance of new lesions or an increase of at least 20% in lesion diameter. Stable disease was defined as changes not meeting criteria for either regression or progression. Surgically resected lesions were classified as complete regression for analytical purposes; absence of recurrence was considered stable disease, whereas regrowth within the resection bed was classified as progression.

## 3. Results

### 3.1. Baseline Characteristics

**Figure 1:**
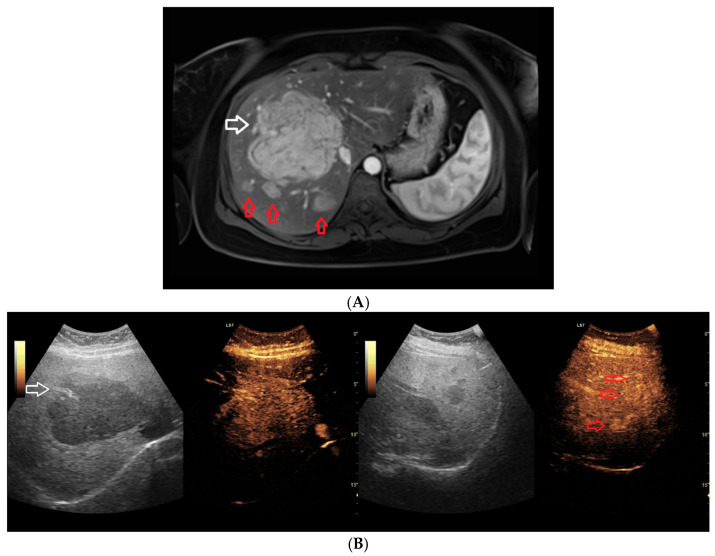
(**A**) Abdominal MRI, T1-weighted sequence after intravenous contrast administration, arterial phase. A large, well-circumscribed focal hepatic lesion with benign imaging characteristics is demonstrated, showing relatively homogeneous arterial contrast enhancement. The white arrow indicates the main focal lesion (in a cross-section with a visible fibrous scar), while the red arrows point to three satellite lesions of the same type. In addition, several smaller accompanying lesions with similar signal characteristics and enhancement patterns are present. (**B**) Contrast-enhanced ultrasound (CEUS) performed for correlation with the MRI findings shown in (**A**). On baseline B-mode imaging, the lesion appears markedly hypoechoic with slightly irregular margins, a presentation that may suggest a malignant character. In the initial contrast-enhanced assessment focused on the central lesion, the satellite lesions previously identified on MRI are not visualized. The left part of the figure shows a white arrow indicating the scar from the cross-section in (**A**), while the satellite lesions remain invisible; they only became visible in a different imaging plane, as shown in the right part of the figure. The main lesion demonstrates a contrast enhancement pattern typical of benign lesions on CEUS. In the later phases of the examination, after a change in the acquisition plane, the satellite lesions become visible; however, they are less conspicuous due to progressive equilibration of contrast enhancement relative to the surrounding liver parenchyma. It should be noted that due to the different imaging axis obtained compared to MRI (resulting from ultrasound imaging limitations), measurement results obtained via CEUS may be inconsistent in some cases, which must be taken into account during the monitoring process.

A total of 41 benign hepatic lesions (28 FNH and 13 HA) were evaluated. One patient had 2 HA lesions, whereas 4 patients had multiple FNH lesions, ranging from 2 to 4 per patient. (Figure 1A,B) The median age of patients in the FNH group was 46 years (IQR, 36.8–54), with a female predominance of 95%. In the HA group, the median patient age was 43 years (IQR, 39–64), and 83.3% of patients were female. Baseline lesion sizes assessed by MRI and CEUS were comparable within each subgroup. In the FNH group, the median maximum lesion diameter was 21 mm on MRI and 21 mm on CEUS, whereas in the HA group the corresponding values were 28 mm and 32 mm, respectively. Detailed measurements are provided in Table 1.

### 3.2. Imaging Tumor Size Comparison (MRI vs. CEUS)

In the combined cohort, paired comparisons of MRI and CEUS measurements, including both baseline and follow-up examinations (a total of 92 paired MRI–CEUS measurements), demonstrated no statistically significant difference in the assessment of the largest lesion diameter between imaging modalities when using linear mixed-effects models. The median largest diameter was 25 mm (IQR, 15–40) on MRI and 25 mm (IQR, 18–38) on CEUS (*p* = 0.1331). Similarly, no statistically significant difference was observed for the smallest lesion diameter, with median values of 21 mm (IQR, 14–30) on MRI and 19 mm (IQR, 14–30) on CEUS (*p* = 0.1546). Longitudinal analysis showed no significant differences between MRI and CEUS in the relative change in the sum of lesion diameters between observations (median 100% for both modalities; *p* = 0.3378). All results are presented as medians with interquartile ranges (Table 2). The distribution of follow-up examinations per lesion is summarized in Table 3.

### 3.3. Lesion Size Evaluation During Follow-Up

**Figure 2:**
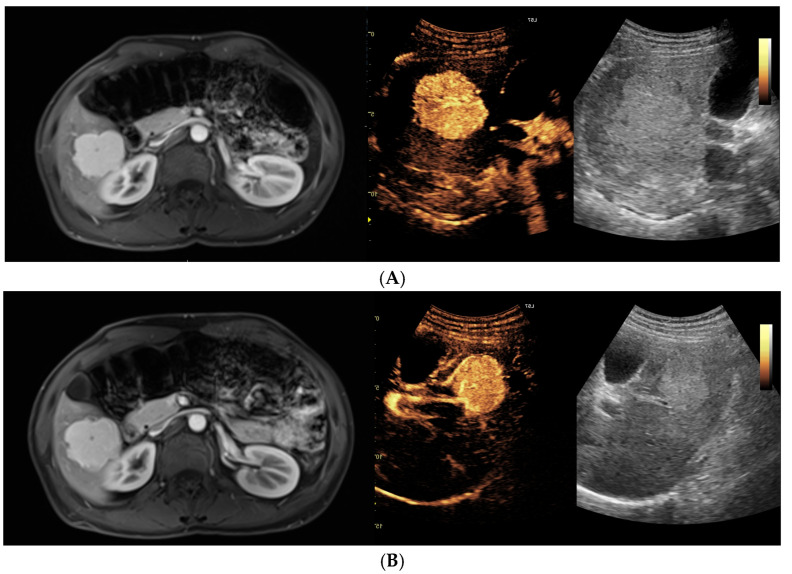
(**A**) Multimodality imaging of a focal hepatic lesion demonstrating high diagnostic concordance between MRI, CEUS, and baseline B-mode ultrasound. The lesion exhibits strong, homogeneous arterial-phase enhancement on both contrast-enhanced modalities, which is indicative of a clearly benign character. Baseline B-mode imaging further supports these findings, showing a homogeneous and well-defined lesion without features suggestive of malignancy, highlighting the excellent correlation between the different imaging techniques. (**B**) Long-term follow-up (48 months) of the hepatic lesion described in (**A**). This panel illustrates the successful use of MRI and CEUS for longitudinal monitoring, showing perfectly consistent results across both modalities. The lesion remains stable in size and morphology, with no criteria for progression. The contrast enhancement pattern remains unchanged and is unequivocally typical of a benign lesion, confirming the high reliability of multimodality correlation for long-term assessment.

**Figure 3:**
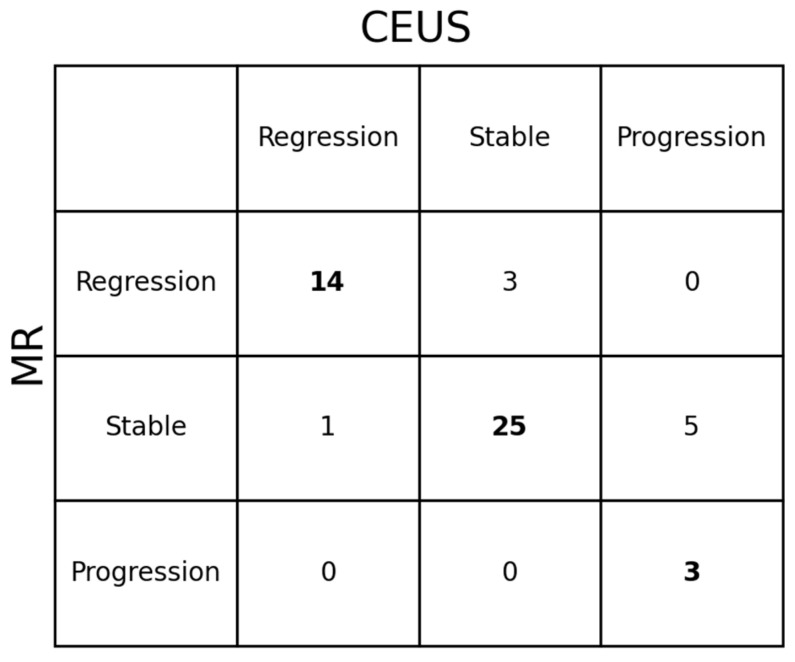
Concordance of liver lesion response classification between contrast-enhanced ultrasound (CEUS) and magnetic resonance imaging (MRI). Rows represent response categories assigned by MRI, and columns represent response categories assigned by CEUS (regression, stable disease, progression). Bold numbers indicate concordant classifications between the two imaging modalities.

Lesion size during follow-up was assessed using RECIST-like criteria, classifying each lesion as showing regression, stable disease, or progression on imaging. MRI and CEUS were concordant in the assigned response category in 42 of 51 paired observations (82.4% concordance). In the majority of cases, both modalities indicated stable disease (Figure 2A,B), whereas fewer lesions met criteria for regression or progression. Discordant assessments occurred in 9 of 51 observations, when lesion response was classified differently by MRI and CEUS; however, overall response evaluation remained comparable between the two imaging modalities (Figure 3). During the follow-up period, 4 lesions underwent surgical resection, including 3 FNH lesions and 1 HA lesion. Among the resected FNH lesions, 1 showed progression with recurrence on follow-up, while 2 underwent elective resection with no recurrence. The resected HA lesion had no post-surgical imaging follow-up available. No additional lesions required surgical intervention, and all remaining unresected lesions were managed non-operatively.

## 4. Discussion

HA and FNH are distinct benign liver lesions; however, they frequently demonstrate overlapping contrast enhancement patterns on both CEUS and MRI, which may complicate the initial diagnostic assessment. In particular, FNH may exhibit enhancement patterns that overlap with those of malignant tumors, making radiologic differentiation between FNH and HA challenging. Up to 20% of FNH lesions lack characteristic imaging features such as a central scar, while certain molecular subtypes of HA may display imaging characteristics closely resembling FNH, including the presence of central scarring and similar contrast enhancement behavior [5,6,15,16,18]. Although FNH is not considered a premalignant condition, in contrast to HA, which carries a small but recognized risk of transformation to HCC [8,9], diagnostic uncertainty on imaging may occasionally result in malignant lesions being initially classified as benign. Rare cases of lesion rupture with hemorrhage and isolated reports of malignant transformation to HCC have been described, and these events are generally attributed to diagnostic inaccuracies, particularly misclassification between HA and FNH. Consistent with this, the literature contains numerous reports of lesions initially diagnosed as FNH that were subsequently reclassified as HA, and vice versa [10,11,12,13,14,18]. For example, in one series, malignant transformation was identified in approximately 5.9% of patients with HA [9]. In this context of overlapping imaging features and documented diagnostic reclassification, the absence of routine imaging surveillance for confidently diagnosed FNH as recommended by EASL guidelines [19] warrants careful consideration. Although these guidelines do not mandate follow up, several reports have suggested that imaging surveillance of both FNH and HA may be considered to document long term stability and to facilitate early detection of atypical changes.

Multiple established risk factors may promote growth in HA, and to a lesser extent in FNH. Estrogen exposure is a key driver, as HA growth is stimulated by estrogen from exogenous sources such as oral contraceptive pills or anabolic steroids, as well as from endogenous overproduction related to obesity [7,24]. Accordingly, women receiving long-term oral contraceptives and individuals undergoing hormonal therapies are at increased risk of HA development and enlargement. Obesity further contributes through increased adipose-derived estrogen production, whereas weight loss or menopause may lead to lesion regression by reducing circulating estrogen levels. HA development has also been associated with metabolic disorders, including glycogen storage disease and hepatocyte nuclear factor 1 alpha maturity-onset diabetes of the young, highlighting the role of metabolic and hormonal dysregulation [7]. Pregnancy represents a distinct high-estrogen state that historically raised concerns regarding HA enlargement or complications [24]. Contemporary practice permits pregnancy in women with known low-risk HA, albeit under strict imaging surveillance due to the recognized risk of accelerated growth during gestation [8,24]. Similarly, intense hormonal stimulation during fertility treatments, including ovulation induction, has been associated with unpredictable behavior of HA, and cases of malignant transformation following such exposure have been reported [25]. In contrast, FNH is generally not hormone driven and lacks clearly defined risk factors for growth. Nevertheless, because of overlapping imaging features, continued diagnostic vigilance is warranted, particularly when risk factors associated with HA are present or when diagnostic certainty is limited [18]. In this context, CEUS alone may not always reliably differentiate HA from FNH. Indeed, one study cautioned that CEUS is less suitable as a stand-alone diagnostic modality for distinguishing between these entities and recommended MRI with liver specific contrast agents for definitive lesion characterization and for the detection of potential malignant transformation [15].

Despite the presence of these risk factors, long-term follow-up studies indicate that most HA and FNH lesions remain stable or regress, with true progression occurring in only a minority of cases. For HA, Vernuccio et al. reported that approximately 90% of solitary HA and 71% of multiple HA remained stable or decreased in size on MRI after a median follow-up of five years following oral contraceptive withdrawal. In the same study, progression of unresected solitary HA was observed in only 16% of cases [9]. Another cohort evaluating HA after cessation of oral contraceptives demonstrated regression or stability in 98.7% of lesions, including complete resolution in approximately 5%, partial regression in 37%, stability in 56%, and progression in only 1.3% [7]. During pregnancy, HA behavior appears more variable but remains predominantly benign. In a retrospective analysis of 73 untreated pregnancies, 53% of lesions were stable, 15% regressed, and 32% demonstrated growth [24], while a prospective study of 51 pregnancies reported stability in 53%, regression in 22%, and growth in 25% of cases [11]. Following partial resection, residual HA rarely progress; a multicenter analysis reported new or enlarging lesions in only 18 of 134 follow-ups, with the majority of lesions remaining stable or regressing [2]. FNH generally follows an even more indolent course. A 9-year follow-up study involving 216 women demonstrated size changes in only 2.9% of lesions [26]. In another series of 167 FNH lesions, interval growth was observed in 15%, regression in 8%, and stability in 77% of cases [27]. Similarly, long-term sonographic follow-up showed that approximately 71% of FNH lesions remained stable, only 3% progressed, and 26% regressed, with complete disappearance reported in 17.6% [28]. More recent data confirm that more than 80% of FNH lesions remain stable or decrease in size during extended follow-up [29]. Collectively, these findings indicate that both HA and FNH typically exhibit stable or slow-growing behavior, with spontaneous regression occurring in a substantial proportion of cases and clinically significant progression remaining uncommon in the absence of high-risk features.

The findings of the present study are consistent with the existing literature. Baseline lesion diameters measured using CEUS and MRI were largely comparable. However, a statistically significant difference was observed in the assessment of the largest lesion diameter, despite identical median values for both modalities (25 mm, *p* = 0.1331). No statistically significant differences were identified for the shortest lesion diameter. During follow-up, lesions remained predominantly stable, with a median size change of 0%, which aligns with previous reports indicating stability or regression in more than 80% of HA and FNH cases [9,27,28,29]. When evaluated using RECIST-like criteria, CEUS and MRI demonstrated high concordance in response classification, with agreement in 42 of 51 follow-up assessments (82.4%). This finding indicates comparable performance of both modalities in detecting lesion behavior over time. The absence of malignant transformation and the limited lesion growth observed in this cohort further support the benign natural history described in earlier studies [9,30]. Minor discrepancies between CEUS and MRI were noted in follow-up response classification. In 3 cases, lesions were classified as stable on CEUS but regressive on MRI, whereas in 1 case regression was identified on CEUS and stability on MRI. Additionally, CEUS classified 5 lesions as progressive that were considered stable on MRI. Importantly, in all cases where progression was concordantly observed by both modalities, the findings were unequivocal. However, the 5 cases of “pseudo-progression” identified only by CEUS were not confirmed by concurrent MRI, which remained the reference standard for stability. These discrepancies highlight a critical limitation regarding the intra-modality reproducibility of CEUS in longitudinal monitoring. While interval percentage changes were calculated within each modality, the lack of standardized, reproducible orthogonal planes in CEUS makes it susceptible to measurement variability between sequential examinations. Unlike MRI, where imaging planes are precisely replicated, CEUS is influenced by the acoustic window, respiratory phase, and changes in patient body habitus (e.g., increased adipose tissue). Such factors may force the operator to adopt a more oblique scanning plane compared to the baseline study, leading to a technical overestimation of the diameter that can exceed the 20% threshold for progression. Consequently, CEUS measurements may occasionally be obtained in partially oblique planes, which can result in subtle overestimation of maximal lesion diameters. This technical variability should be considered when interpreting small inter-modality differences, especially in longitudinal follow-up, where minor measurement deviations may influence response classification. In this context, it is important to emphasize the central role of MRI as the baseline imaging modality for the entire monitoring process. This is particularly relevant in patients with multiple lesions and in cases where lesions are located in anatomically challenging regions, such as subdiaphragmatic areas, where ultrasonographic visualization may be limited. Establishing an accurate MRI baseline enables consistent follow-up and facilitates reliable interpretation of subsequent CEUS examinations. CEUS allows for detailed morphological assessment of focal liver lesions and enables precise evaluation of enhancement patterns, particularly arterial phase wash-in, which typically becomes apparent approximately 20 s after contrast agent administration. Therefore, it is essential that the ultrasonographer is fully aware of the exact lesion location prior to contrast injection, rather than attempting to identify the lesion during the dynamic examination. Within this short time window, lesion echogenicity may rapidly become isoechoic relative to the surrounding liver parenchyma, potentially impairing reliable identification and measurement. Accurate lesion localization based on baseline MRI is thus critical for optimal CEUS performance, ensuring correct targeting during the arterial phase and improving the reliability of longitudinal lesion assessment.

The high level of agreement between CEUS and MRI observed in this study underscores the clinical utility of CEUS as a surveillance modality. CEUS offers several practical advantages, including the use of microbubble contrast agents with a favorable safety profile, permitting repeated examinations without cumulative risk. This is particularly relevant during pregnancy, as gadolinium-based MRI contrast agents cross the placental barrier and their long-term fetal safety remains uncertain [5,6,31]. In contrast, CEUS contrast agents do not cross the placenta, and no fetal enhancement has been observed, supporting their safety in pregnant patients [31]. Furthermore, CEUS is widely available, cost-effective, and suitable for point-of-care use, facilitating frequent follow-up when clinically indicated. Previous studies suggest that CEUS may serve as a first-line modality for FNH evaluation and as a follow-up tool for lesions previously characterized by MRI, with MRI reserved for cases demonstrating interval growth or changes in enhancement patterns [15,18].

To our knowledge, this study represents the first direct comparison of CEUS and contrast-enhanced MRI for the longitudinal monitoring of HA and FNH. While the study is limited by its single-center design and relatively small cohort size, the findings must be interpreted in the context of major reference studies comparing CEUS and MRI, specifically the work by Bröker et al. and Swensson et al. These studies differ fundamentally from ours in design; they assessed diagnostic accuracy for the initial differentiation of FNH and HA in a cross-sectional setting, whereas our study focused on longitudinal surveillance. Bröker et al. analyzed a significantly larger cohort (156 patients), including histopathological confirmation in discordant cases, demonstrating that while CEUS shows fair agreement with MRI, liver-specific contrast-enhanced MRI remains the superior diagnostic reference standard. Similarly, Swensson et al. highlighted the limitations of CEUS in a prospective trial, noting that CEUS accuracy approaches that of hepatobiliary MRI only for lesions larger than 20 mm, while smaller lesions remain diagnostically challenging [15,16]. While these studies rightly suggest that MRI is preferable for initial definitive characterization, particularly for small or ambiguous nodules, our longitudinal data indicate that once this baseline diagnosis is secured, CEUS performs comparably to MRI in detecting interval growth or morphological stability. Consequently, this supports the utility of CEUS as a practical modality for long-term monitoring. Additional limitations of the present study relate to specific technical and methodological challenges. First, the characterization of subcentimeter lesions (5–10 mm) presents inherent diagnostic difficulties; however, these were included as satellite nodules of larger, definitive index lesions to evaluate whether their enhancement patterns might demonstrate features typical of malignancy over time. Furthermore, we acknowledge that grouping FNH and HCA represents a methodological simplification given their distinct biological natures. This approach was adopted because both lesion types exhibit similar enhancement curves that clearly differentiate them from malignant masses, which served as the technical basis for our analysis [5,6]. Moreover, differentiating these two entities remains a significant challenge in routine clinical practice, as discussed above. Nevertheless, the high level of concordance observed between CEUS and MRI supports the potential role of CEUS as a reliable imaging modality for follow-up in this setting. These findings suggest that CEUS may serve as an alternative surveillance tool in appropriately selected patients, although further validation in larger, multicenter prospective studies is required before these results can be translated into broader clinical follow-up strategies.

## 5. Conclusions

Once a diagnosis of FNH or HA has been established using contrast enhanced MRI as the baseline reference for diagnosis and subsequent monitoring, and the lesion shows no features warranting surgical intervention or after removal of potential factors that may promote lesion growth, CEUS may be used as the sole imaging modality for follow up. This approach is supported by the comparable enhancement characteristics of both lesion types on CEUS and MRI, as well as the similar accuracy of lesion size assessment and lesion classification across modalities. Given its lower cost, wider availability, and better patient tolerability, CEUS represents a practical alternative to MRI for routine surveillance. If CEUS demonstrates a clinically meaningful change in lesion size or an alteration in enhancement patterns suggestive of malignant transformation, contrast enhanced MRI should be performed to confirm the findings, preserving its role as the diagnostic gold standard.

## Figures and Tables

**Table 1:** Demographic characteristics and lesion size measurements in focal nodular hyperplasia (FNH) and hepatocellular adenoma (HA) cohorts. Lesion diameters were assessed using magnetic resonance imaging (MRI) and contrast-enhanced ultrasound (CEUS). SD, standard deviation; IQR, interquartile range.

	FNH *n* = 28	HA *n* = 13
Age—Median (IQR)	46 (36.8–54)	43 (39–64)
Sex		
Female	19 (95.0%)	10 (83.3%)
Male	1 (5.0%)	2 (16.7%)
Higher diameter MRI—Median (IQR) [mm]	21 (14–35.5)	28 (23–41)
Higher diameter CEUS—Median (IQR) [mm]	21 (15–36)	32 (24.5–42)
Lower diameter MRI—Median (IQR) [mm]	15 (11–28)	23 (21–38.5)
Lower diameter CEUS—Median (IQR) [mm]	16 (12–29.5)	20 (18–37)

**Table 2:** Descriptive statistics of tumor dimensions and changes in tumor size. Data are presented as medians with interquartile ranges (IQR). To increase statistical power, hepatocellular adenoma (HA) and focal nodular hyperplasia (FNH) were analyzed as a single study group, as both entities exhibit comparable enhancement patterns on magnetic resonance imaging (MRI) and contrast-enhanced ultrasound (CEUS). Tumor measurements were obtained using MRI and CEUS. Statistical comparisons between imaging modalities were performed using linear mixed-effects models to account for repeated measurements and intra-lesion correlation. A *p*-value < 0.05 was considered statistically significant.

	MRI	CEUS	*p*
Higher diameter			
Median [mm]	25	25	0.1331
IQR [mm]	15–40	18–38	
Min–Max [mm]	5–102	5–105	
Lower diameter			
Median [mm]	21	19	0.1546
IQR [mm]	14–30	14–30	
Min–Max [mm]	4–93	5–80	
Relative change in sum of diameters between observations [%]			
Median [mm]	100	100	0.3378
IQR [mm]	99–101	99–101	
Min–Max [mm]	58–138	51–143	

**Table 3:** Detailed structure of the study dataset and follow-up characteristics.

**Total number of lesions followed**	41
Baseline measurements (one per lesion)	41
Total follow-up measurements	51
**Follow-up examinations per lesion**	
Mean	2.42
Range (Min–Max)	1–6
**Follow-up intervals**	
Mean	12.8 months
Maximum interval	52.1 months
**Total Follow-up duration**	
Median Follow-up duration	8.5 months
Maximum Follow-up duration	72.6 months

## Data Availability

The data presented in this study are available on request from the corresponding author.

## Abbreviations

The following abbreviations are used in this manuscript:

ADC	Apparent Diffusion Coefficient
B-mode	Brightness mode
CEUS	Contrast-Enhanced Ultrasound
DWI	Diffusion-Weighted Imaging
EASL	European Association for the Study of the Liver
FNH	Focal Nodular Hyperplasia
HA	Hepatocellular Adenoma
HCC	Hepatocellular Carcinoma
IQR	Interquartile Range
MI	Mechanical Index
MRI	Magnetic Resonance Imaging
RECIST	Response Evaluation Criteria in Solid Tumors
SD	Standard Deviation
T1-w	T1-weighted
T2-w	T2-weighted
US	Ultrasound
